# P58 Drug resistance of *Mycobacterium tuberculosis* isolated from bone tissue of patients with tuberculous spondylitis and coinfections of HIV/HCV: identification of a high-risk group for MDR TB

**DOI:** 10.1093/jacamr/dlaf230.065

**Published:** 2025-12-04

**Authors:** T V Minogina, L S Lavrenchuk, D V Vakhrusheva, S N Skornyakov

**Affiliations:** NMRC for Phthisiopulmonology and Infectious Diseases, Ministry of Health of Russia, Yekaterinburg, Russia; NMRC for Phthisiopulmonology and Infectious Diseases, Ministry of Health of Russia, Yekaterinburg, Russia; NMRC for Phthisiopulmonology and Infectious Diseases, Ministry of Health of Russia, Yekaterinburg, Russia; NMRC for Phthisiopulmonology and Infectious Diseases, Ministry of Health of Russia, Yekaterinburg, Russia

## Abstract

**Background:**

Tuberculous spondylitis (TS, Pott’s disease) accounts for up to 50% of extrapulmonary TB cases and presents a significant clinical challenge due to destructive spinal involvement, frequent neurological complications and difficulties in detecting *Mycobacterium tuberculosis* (Mtb) in bone tissue. Molecular diagnostics have improved pathogen detection; however, cases with concomitant HIV andHCV infections remain problematic. These conditions, marked by immunosuppression, systemic inflammation, endothelial dysfunction and altered host-pathogen interactions, are linked to atypical TS progression, rapid disease course and reduced treatment efficacy. Analysing Mtb genome mutations is critical for rational chemotherapy selection, especially when rapid drug susceptibility results are unavailable.

**Objectives:**

To define the mutation patterns and frequency associated with resistance to isoniazid, rifampicin, fluoroquinolones and aminoglycosides in Mtb isolated from bone tissue of TS patients coinfected with HIV and HCV, and to justify including this group in the high-risk category for MDR TB.

**Methods:**

A retrospective and prospective study included 300 patients with a confirmed diagnosis of TS, who were observed from 2016 to 2021 in a specialized clinic. Patients were grouped as follows: 20% without viral infections, 8% infected only with HCV, 6% with HIV alone and 66% with combined HIV+HCV infection. Surgical bone specimens were analysed using microscopy, culture on solid media and molecular-genetic techniques (PCR/PCR reverse hybridization). When detecting MBT DNA in amounts insufficient for identifying mutations, a repeat study was conducted. Among 324 PCR-positive cases, 267 samples contained sufficient DNA for mutation detection. Key genetic loci examined included *rpoB*, *katG*, *gyrA*/*gyrB*, *rrs* and *eis*. Results were correlated with patients’ viral infection status.

**Results:**

PCR showed the highest sensitivity, detecting Mtb DNA in 83% of bone samples, compared to 28% with microscopy and 24% with culture. Resistance-related mutations were common: *rpoB* mutations (rifampicin resistance) were found in 72.3% of strains, predominantly the S531L substitution (86.5%); *katG* mutations (isoniazid resistance) in 79.4%, mainly S315T (96.2%). Fluoroquinolone resistance (*gyrA*/*gyrB*) was present in 31.6% of samples, aminoglycoside resistance (*rrs*, *eis*) in 51.6%. HIV infection increased the risk of MDR Mtb 4.16-fold (95% CI 2.11–7.67) and HCV 2.29-fold (95% CI 1.36–4.86). Patients with dual HIV+HCV infection showed the lowest proportion of drug-susceptible strains and the highest frequency of pre- XDR Mtb. Overall, the likelihood of detecting at least MDR Mtb in this subgroup was nearly four times higher than in patients without viral infections.

**Conclusion:**

Molecular diagnostics outperform traditional bacteriology in detecting the TS pathogen and its drug resistance mutations. The high frequency of *rpoB* and *katG* mutations, combined with changes in *gyrA*/*gyrB*, *rrs* and *eis*, confirms a significant risk of MDR/pre-XDR Mtb in TS patients with HIV/HCV coinfections. Given the time required for results and low culture sensitivity, personalized therapy based on molecular-genetic analysis of bone specimens is optimal. When such data are unavailable, empirical treatment targeting MDR/pre-XDR Mtb is justified. Molecular diagnostics thus serve both as a rapid detection tool and a key to stratifying patients by MDR Mtb risk for informed personalized or empirical treatment decisions.

